# The Genetic Diversity of *Rickettsiella* Symbionts in *Ixodes ricinus* Throughout Europe

**DOI:** 10.1007/s00248-021-01869-7

**Published:** 2021-09-28

**Authors:** Aitor Garcia-Vozmediano, Laura Tomassone, Manoj Fonville, Luigi Bertolotti, Dieter Heylen, Nannet D. Fabri, Jolyon M. Medlock, Ard M. Nijhof, Kayleigh M. Hansford, Hein Sprong, Aleksandra I. Krawczyk

**Affiliations:** 1 Department of Veterinary Sciences, University of Turin, L.go Braccini, 2, 10095 Grugliasco, TO Italy; 2 Centre for Infectious Disease Control, National Institute for Public Health and the Environment (RIVM), Antonie van Leeuwenhoeklaan 9, 3720 BA Bilthoven, The Netherlands; 3 Eco-Epidemiology Group, Department of Biomedical Sciences, Institute of Tropical Medicine, Antwerp, Belgium; 4 Evolutionary Ecology Group, Department of Biology, University of Antwerp, Wilrijk, Belgium; 5 Interuniversity Institute for Biostatistics and Statistical Bioinformatics, Hasselt University, Diepenbeek, Belgium; 6 Department of Wildlife, Fish, and Environmental Studies, Swedish University of Agricultural Sciences, 901 83 Umeå, Sweden; 7 Department of Population Health Sciences, Faculty of Veterinary Medicine, Utrecht University, Yalelaan 7, 3584 CL Utrecht, The Netherlands; 8 Infections Medical Entomology & Zoonoses Ecology, Public Health England, Porton Down, UK; 9 Institute for Parasitology and Tropical Veterinary Medicine, Freie Universität Berlin, Robert-von-Ostertag-Str. 7-13, 14163 Berlin, Germany; 10 Laboratory of Entomology, Wageningen University and Research Centre, Wageningen, The Netherlands

**Keywords:** *Rickettsiella*, Tick-borne bacteria co-infection, Facultative symbionts, *Ixodes ricinus* ecology, Tick-borne pathogens

## Abstract

*Rickettsiella* species are bacterial symbionts that are present in a great variety of arthropod species, including ixodid ticks. However, little is known about their genetic diversity and distribution in *Ixodes ricinus*, as well as their relationship with other tick-associated bacteria. In this study, we investigated the occurrence and the genetic diversity of *Rickettsiella* spp. in *I. ricinus* throughout Europe and evaluated any preferential and antagonistic associations with *Candidatus* Midichloria mitochondrii and the pathogens *Borrelia burgdorferi* sensu lato and *Borrelia miyamotoi*. *Rickettsiella* spp. were detected in most *I. ricinus* populations investigated, encompassing a wide array of climate types and environments. The infection prevalence significantly differed between geographic locations and was significantly higher in adults than in immature life stages. Phylogenetic investigations and protein characterization disclosed four *Rickettsiella* clades (I–IV). Close phylogenetic relations were observed between *Rickettsiella* strains of *I. ricinus* and other arthropod species. Isolation patterns were detected for Clades II and IV, which were restricted to specific geographic areas. Lastly, although coinfections occurred, we did not detect significant associations between *Rickettsiella* spp. and the other tick-associated bacteria investigated. Our results suggest that *Rickettsiella* spp. are a genetically and biologically diverse facultative symbiont of *I. ricinus* and that their distribution among tick populations could be influenced by environmental components.

**Supplementary Information:**

The online version contains supplementary material available at 10.1007/s00248-021-01869-7.

## Introduction

Ticks interact with multiple biotic and abiotic factors that modulate the success of their survival and development. Environmental factors — such as climate, habitat type, and host availability — are among the most limiting drivers for ticks, determining their geographical spread and abundance [1, 2]. Moreover, ticks are involved in commensal, mutualistic, and parasitic relationships with a wide range of microbes, including bacteria, viruses, and protozoa, which can cause infectious diseases in humans and animals. Together, they compose the so-called tick microbiota [3].

Among members of tick microbiota, tick-borne pathogens have sparked the greatest interest, given their worldwide impact on public and veterinary health [4–7]. However, recent advances in molecular techniques enabled the identification of complex bacterial communities residing in ticks, including symbiotic bacteria that display an important role in tick ecology and physiology [8]. In fact, some of these symbiotic bacteria are considered essential for tick survival since their absence reduces the reproductive fitness of ticks [9]; others supplement the unbalanced diet of ticks by biosynthesizing vitamins that are scarce in vertebrate blood [10, 11]. Microbial communities within ticks have been hypothesized to modulate the occurrence and diversity of tick-borne pathogens and consequently determine their transmission to vertebrate hosts [8].

*Ixodes ricinus* is the most widespread tick species in Europe and the primary vector of *Borrelia burgdorferi* sensu lato, the causative agent of Lyme borreliosis [12]. It also transmits *Borrelia miyamotoi*, an emerging human pathogen belonging to the relapsing fever group borreliae [13]. In the past years, several studies have explored *I. ricinus* microbiota, identifying different heritable bacteria. Among these, *Candidatus* Midichloria mitochondrii (hereafter *M. mitochondrii*) is one of the most abundant symbionts and widespread among several tick species. It is mainly located within the mitochondria of host ovary cells [14, 15], however, it has been also identified in salivary glands and Malpighian tubules, suggesting that it might play different roles in tick physiology [16]. Although there is evidence for the presence of *M. mitochondrii* DNA in vertebrate hosts of ticks, it remains unclear whether its presence in vertebrates may cause disease or may facilitate horizontal transmission [17].

Other heritable bacteria residing in *Ixodes* ticks are obligate intracellular bacteria of the genus *Rickettsiella* [18–22]. *Rickettsiella* spp. have been also associated with other arthropods, including insects, arachnids, and crustaceans [23–29]. Depending on the arthropod host, the infection by some *Rickettsiella* species was shownto hinder the hosts’ development and survival [23], or manipulate host reproduction [30]. Other *Rickettsiella* spp. have shown a biological significance for the survival of their hosts by manipulating the host attractiveness to predators and parasitoids [31].

Relatively few studies have so far reported on *Rickettsiella* infection in *I. ricinus* [20, 32–35], and its biological role in this important vector is entirely unknown. In the present study, we examined European *Ixodes* ticks for the presence of *Rickettsiella* spp. and determined its genetic diversity, with a focus on *I. ricinus.* Moreover, we evaluated possible associations of *Rickettsiella* spp. with *M. mitochondrii* and the pathogens *B. burgdorferi* s.l. and *Borrelia miyamotoi*.

## Materials and Methods

### Tick Sampling

To evaluate the presence of *Rickettsiella* symbionts in *I. ricinus* populations, we studied a total of 4941 questing ticks from different latitudes across Europe (Appendix), which were morphologically identified to species level [36]. Northwestern and central Europe were represented by 2033 *Ixodes ticks* collected in green areas of Antwerp (Belgium; [37]), 999 *I. ricinus* from nine different forest sites across the Netherlands and 66 *I. ricinus* nymphs from Güterfelde, north-eastern Germany, collected by dragging in April 2019. We also included 240 *I. ricinus* collected in two different areas from the southern United Kingdom. Northern latitudes were represented by tick populations collected in September 2018 in forested areas in Södermanland county, Sweden (*n* = 235). Finally, Southern Europe accounted for three mountain areas located in north-western and central Italy (*n* = 1,368) as previously described [38–40]. Furthermore, some specimens of questing *Ixodes frontalis* (*n* = 4) and *Ixodes hexagonus* (*n* = 6) ticks from Belgium were also included in this study.

### Detection of* Rickettsiella* Symbionts and Other Tick-Borne Bacteria

DNA extraction from ticks of most locations was performed using ammonium hydroxide [41], except for the ticks from northwestern and central Italy from which tick DNA was extracted using DNAzol reagent (Life Technologies LTD, Warrington, UK) [40] and the QIAGEN DNeasy tissue kit (Qiagen, Hilden, Germany) [38], respectively.

All ticks were screened by multiplex polymerase chain reaction (qPCR) assays targeting the *ospA and flaB genes* of *B. burgdorferi* s.l. and the *flaB* gene of *B. miyamotoi* as previously described in [42, 43]. To detect the tick symbionts *Rickettsiella* spp. and *M. mitochondrii* a multiplex qPCR protocol was designed using primers and probes described in Table 1. A sample was considered positive for *Ricketsiella* if at least one of the two targets, either *gidA* or *sucB*, was positive. Positive qPCR-*Rickettsiella* samples were subjected to end-point conventional PCR targeting a 786-bp fragment of *gidA* gene with primers previously described in [44].

**Table 1:** Marker genes with qPCR primers and probes used for amplification

Tick symbiont	Gene acronym	Function of gene product	PCR primers	Length of amplified sequences (bp)
***Rickettsiella* spp.**	***gidA***	Glucose inhibited cell division protein A	fwd: 5′- TGT AAT CCT TGA GTC TGA TCG T	196
			rev: 5′- CAA ACC GAT ATG AAT TTT TCC GG	
			probe: 5′- ATTO520-TAG TTG GTG TGG TAA CGC AAA TGG GGT-BHQ2	
	***sucB***	Dihydrolipoamide succinyl-transferase component E2	fwd: 5′- GAT CAA CCC TCT CAA TCA GC	76
			rev: 5′- GCC AAA TGG GTG TCA CTA T	
			probe: 5’- ATTO647- CAC CCG TCG CAG AAA AAA CTA AAC CTG-BHQ2	
***Midichloria mitochondrii***				
	***gyrB***	Gyrase subunit B	fwd: 5′- CTT GAG AGC AGA ACC ACC TA	145
			rev: 5′- CAA GCT CTG CCG AAA TAT CTT	
			probe: 5′- ATTO424- GAG GGC GGA GTC AAA GAA TTT GTC CAC G-BHQ1	

All qPCRs were carried out on a LightCycler 480 (Roche Diagnostics Nederland B.V, Almere, the Netherlands) in a final volume of 20 μl with iQ multiplex Powermix, 3 μl of sample, primers with end concentration of 0.2 μM, and probes. Positive plasmid controls and negative water controls were used on every plate tested. Cycling conditions included an initial activation of the iTaq DNA polymerase at 95 °C for 5 min, followed by 60 cycles of a 5-s denaturation at 95 °C followed by a 35-s annealing‐extension step at 60 °C (ramp rate 2.2 °C s^−1^ and a single point measurement at 60 °C) and a final cooling cycle of 37 °C for 20 s. To minimize contamination and false-positive samples, the DNA extraction, PCR mix preparation, sample addition, and qPCR analyses were performed in separate air-locked laboratories.

### Statistical Analyses

Tick infection and coinfection prevalence and 95% binomial confidence intervals were calculated using RStudio software v1.1.463 [45]. Moreover, we compared the frequency of tick-borne bacterial infections and coinfections among the European countries investigated, including sampling areas within the single regions, as well as between tick life stages (nymphs and adults) using Pearson’s chi-squared test. Finally, we used the Kappa (*κ*) statistic to assess the association by quantifying coinfections of *Rickettsiella* spp. with tick-borne pathogens (*B. burgdorferi* s.l. and *B. miyamotoi*) and *M. mitochondrii* [46].

### Phylogenetic Analyses

Forward and reverse nucleotide sequences for each *Rickettsiella gidA* amplicon were accurately assembled and manually corrected into final nucleotide sequences of 723 bp using Geneious Prime (version 2020.0.4) software. These were aligned to available reference *gidA* sequences from different arthropod species, including ticks from North America, such as *Ixodes angustus* (GenBank accession n. HG792871), *Ixodes kingi* (HG792872), *Ixodes sculptus* (HG792873) and *Ixodes woodi* (JQ070345), other arachnid species (KU597421) and several insects belonging to the orders Coleoptera and Isopoda (JQ679309, JX406182, JN565687-89–91, JF288927)*.* A *Coxiella burnetii* (AE016828.3) sequence was used as an outgroup given its evolutionary relationship with *Rickettsiella* spp. The nucleotide alignment was performed using ClustalW [47] respecting the coding frame. This method avoids nucleotide sequence misreading based on the conversion of nucleotide sequences into their corresponding peptide sequences which, in turn, will be eventually translated back into the nucleotide sequences they have originated from [48]. Nucleotide and amino acid diversities were calculated among all the analysed samples and expressed as percentage. Nucleotide alignment as well as the amino acid variable positions was evaluated in order to investigate the possible population structure. We assessed the phylogenetic relationships among *Rickettsiella* positive samples and reference sequences using a MCMC Bayesian approach by using the GTR substitution model [49] embedded in MrBayes software v3.2.7a [50]. The resulting consensus tree was viewed and edited using FigTree v1.4.4 [51].

## Results

### Occurrence of* Rickettsiella* spp. in Different European Regions

*Rickettsiella* spp. was widespread in most of the *I. ricinus* populations, although its occurrence between geographic locations varied (Pearson’s Chi-squared test, *p* < 0.001). Regions from western Europe showed the highest infection rates, except for the tick population from Germany, in which the bacterium was not detected at all. Tick populations from Belgium reached a prevalence of nearly 90%, followed by around 60% and 50% of infection observed in populations from the Netherlands and UK, respectively (Table 2). We conversely recorded the lowest infection rates in *I. ricinus* ticks from northern and southern latitudes (Sweden and Italy), with less than 20% prevalence (Table 2). This variability was also evident within the single regions in the countries where different areas were sampled (Table 3); infection rates differed significantly among study sites in the Netherlands (*p* < 0.001), UK (*p* < 0.01) and Italy (*p* < 0.001). The bacterium occurred in all Dutch tick populations (*n* = 9), with a prevalence ranging between 16.1% (95% CI = 7.6–28.3) and 99.0% (95% CI = 94.3–100). Moderate infection rates were observed in the two tick populations from the UK, with 38.3% (95% CI = 29.6–47.6) and 57.5% (95% CI = 48.1–66.5) of infected ticks. The symbiont occurred in a low proportion of *I. ricinus* ticks collected in mountain areas from Italy, and only in one out of three locations investigated (Table 3; 11.8% positives out of 296 *I. ricinus* tested). Lastly, *Rickettsiella* DNA was also detected in all four *I. frontalis* and six *I. hexagonus* specimens tested from Belgium.

**Table 2:** Infection prevalence (%) and 95% CIs of *Borrelia burgdorferi* s.l., *Borrelia miyamotoi*, *Midichloria mitochondrii*, and *Rickettsiella* spp. in *Ixodes ricinus* by region and tick-life stage

Country	Tick life stage tested (*n*)	Pathogen infection (%) [95% CI]		Symbiont infection (%) [95% CI]	
		*B. burgdorferi s.l*	*B. miyamotoi*	*M. mitochondrii*	*Rickettsiella* spp*.*
Belgium	Overall infection	18.4 [16.7–20.1]	1.6 [1.1–2.3]	77.9 [75.7–79.4]	88.6 [87.2–90.0]
	L	-	-	-	-
	N (*n* = 1,771)	16.8 [15.1–18.6]	1.5 [1.0–2.1]	77.7 [75.7–79.6]	87.6 [86.0–89.1]
	F (*n* = 126)	30.9 [23.0–39.8]	3.2 [0.9–7.9]	98.4 [94.4–99.8]	94.4 [88.9–97.7]
	M (*n* = 136)	28.7 [21.2–37.1]	2.2 [0.5–6.3]	61.8 [53.0–70.0]	96.3 [91.6–98.8]
UK	Overall infection	7.9 [4.8–12.1]	0.8 [0.1–3.0]	48.7 [42.3–55.3]	47.9 [41.4–54.4]
	L	-	-	-	-
	N (*n* = 218)	7.3 [4.2–11.6]	0.9 [0.1–3.3]	45.0 [38.2–51.8]	45.0 [38.2–51.8]
	F (*n* = 22)	13.6 [2.9–34.9]	0 [0–15.4]	86.4 [65.1–97.1]	77.3 [54.6–92.2]
	M	-	-	-	-
Germany	Overall infection	6.1 [1.7–14.8]	1.5 [0.03–8.2]	36.4 [24.9–49.1]	0 [0–0.5]
	L	-	-	-	-
	N (*n* = 66)	6.1 [1.7–14.8]	1.5 [0.04–8.2]	36.4 [24.9–49.1]	0 [0–0.5]
	F	-	-	-	-
	M	-	-	-	-
Italy	Overall infection	15.2 [13.3–17.2]	0.6 [0.2–1.2]	68.9 [66.4–71.4]	3.1 [2.2–4.1]
	L	-	-	-	-
	N (*n* = 1,351)	14.5 [12.6–16.5]	0.5 [0.2–1.1]	69.4 [66.8–71.9]	2.5 [1.7–3.5]
	F (*n* = 17)	38.1 [23.6–54.4]	1.7 [0.04 – 8.4]	54.8 [38.7–70.2]	21.4 [10.3–36.8]
	M	-			
The Netherlands	Overall infection	12.3 [9.9–15.2]	1.6 [0.9–2.6]	73.6 [63.0–82.4]*	61.9 [58.0–65.7]
	L (*n* = 367)	0.3 [0.0 – 1.5]	1.4 [0.4–3.2]	84.2 [80.1 – 87.8]	36.8 [31.8 – 41.9]
	N (*n* = 589)	11.6 [9.1–14.4]	1.9 [0.9–3.3]	74.4 [63.2–83.6]	62.4 [58.4–66.3]
	F (*n* = 21)	9.5 [1.2 – 30.4]	0 [0–16.1]	100 [54.1–100]	57.1 [34.0–78.2]
	M (*n* = 22)	36.4 [17.2–59.3]	0 [0–15.4]	0 [0–84.2]	54.5 [32.2–75.6]
Sweden	Overall infection	23.0 [17.8–28.9]	0.9 [0.1–3.0]	62.1 [55.6–68.4]	16.2 [11.7–21.5]
	L	-	-	-	-
	N (*n* = 216)	23.6 [18.1–29.8]	0.9 [0.1–3.3]	63.0 [56.1–69.4]	14.8[10.4–20.3]
	F (*n* = 10)	10.0 [0.3–44.5]	0 [0–30.8]	100 [66.3–100]	20.0 [2.5–55.6]
	M (*n* = 9)	22.2 [2.8–60.0]	0 [0–30.8]	0 [0–30.8]	33.3 [7.5–70.1]

^*^ Only 87 out of 999 ticks were tested for *M. mitochondrii*

*Note*: Names of tick life stage categories were abbreviated: L (larvae), N (nymphs), F (female) and M (male); “-” indicates no specimens were tested for the corresponding tick life stage category

**Table 3:** *Rickettsiella* spp. infection prevalence (%) and 95% CIs in *Ixodes ricinus* stratified by location

Region	Location	N ticks tested	% [95% CI]
The Netherlands	Amsterdamse Waterleiding Duinen	349	52.7 [47.3–58.1]
	Bergherbos	56	16.1 [7.6–28.3]
	Buunderkamp	171	21.6 [15.7–28.6]
	Duin en Kruidberg	96	99.0 [94.3–99.7]
	Deelerwoud	64	31.3 [20.2–44.1]
	Herperduin	48	18.8 [8.9–32.6]
	Kremboong	88	88.6 [80.1–94.4]
	Stameren	48	37.5 [24.0–52.6]
	Zwanemeerbos	80	95.0 [87.7–98.6]
Italy	Alpi Cozie Natural Park	785	0 [0–1.0]
	Aosta Valley	296	11.8 [8.4–16.1]
	Tuscany	287	0 [0–1.0]
Germany	Güterfelde	66	0 [0–1.0]
UK	Salisbury	120	57.5 [48.4–66.5]
	Dartmoor	120	38.3 [29.6–47.6]
Sweden	Södermanlands	235	16.2 [11.7–21.5]
Belgium	Antwerp	2033	88.6 [87.1–89.9]

### *Rickettsiella* spp. Through *Ixodes ricinus* Ontogeny

Adults (*n* = 389) and nymphs (*n* = 4185) were analysed, as well as a sample of 367 larvae from the Netherlands. Larvae, nymphs, and adult ticks harboured *Rickettsiella* spp*.* (Table 2), but the infection exhibited substantial changes according to tick life stage (*p* < 0.001) and the odds of infection for adults doubled those for nymphs (OR = 2.4, 95% CI = 1.7–3.4). Only in the Netherlands, infection in nymphs slightly prevailed over the adults’ prevalence, but the difference was not statistically significant (*p* = 0.5). Moreover, 36.8% (95% CI = 31.8–41.9) of Dutch larvae tested qPCR-positive for *Rickettsiella* spp. *Rickettsiella* infection was comparable in males and females (Pearson’s chi-squared test, *p* = 0.9), with a prevalence of 80.9% (95% CI = 74.4–86.3) and 81.7% (95% CI = 75.4–87.0), respectively.

### Genetic Diversity of *Rickettsiella* spp.

We successfully obtained 263 *gid*A gene (723 bp) nucleotide sequences from *I. ricinus* from most European regions in the study, and one from *I. frontalis* collected in Belgium. Overall, *gidA* sequences showed a high level of similarity, with 85 of 100 sequences from Belgium, 65/98 from the Netherlands, 37/52 from the UK, 6/8 from Sweden, and 2/5 from Italy having 100% of identity. Given the amount of *gid*A sequences produced and the high degree of similarity observed between them, we selected 115 sequences comprising all nucleotide sequences with different levels of dissimilarity (*n* = 75), and a batch of those sharing 100% of identity (*n* = 40), representative for each European region.

**Fig. 1:**
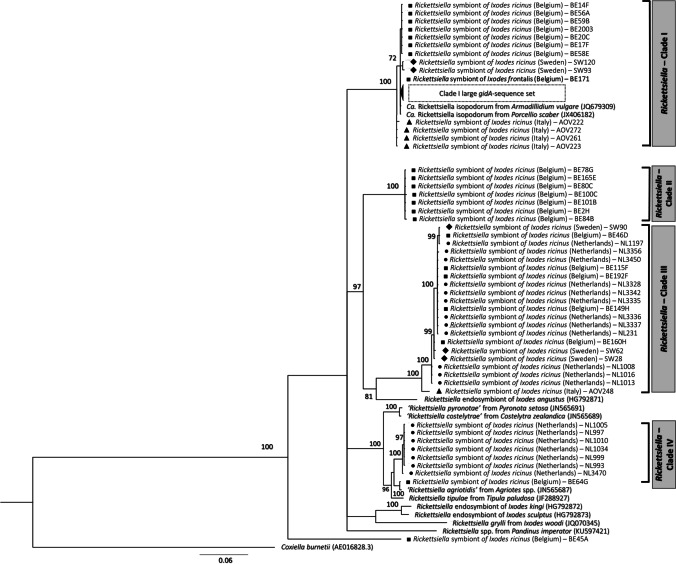
Phylogenetic tree of *gidA* gene of *Rickettsiella* spp. obtained from questing *Ixodes ricinus* populations collected from different European regions. The tree was built by using GTR substitution model (γ = 0.05) and nodal support is given as a posterior probability for Bayesian Inference under percentage format; only probability values above 70 are shown next to the internal nodes. *Coxiella burnetii* was used as the outgroup. Reference sequences are identified by GenBank accession number enclosed in parentheses. Amplicons obtained in the study are indicated with a black symbol representing the region of provenance: Belgium (■), Italy (▲), the Netherlands (●), Sweden (◆), and the UK (▼). A large *gidA* sequence set from *Rickettsiella*-Clade I was removed from the tree for readability purposes, containing a total of 64 sequences identified in *I. ricinus* from Belgium (*n* = 10), the Netherlands (*n* = 28), UK (*n* = 25), and Sweden (*n* = 1)

Phylogenetic tree topology showed a clustering of the *Rickettsiella* population into four well-defined clades (Fig. 1) which, at time, exhibited different frequencies (*p* < 0.001). *Rickettsiella*-Clade I represented the largest group observed comprising 81.7% (95% CI = 76.5–86.2) of the entire population followed by Clade III (12.2%; 95% CI = 8.5–16.8). *Rickettsiella*-Clades II and IV, on the other hand, respectively accounted for 2.7% (95% CI = 1.1–5.4) and 3.1% (95% CI = 1.3–5.9) of the symbiont population, thus representing the least frequent *Rickettsiella* Clades detected in *I. ricinus* ticks. We also identified a *Rickettsiella* spp. from a single *I. frontalis* nymph that clustered within *Rickettsiella*-Clade I and showed 99.6–100% of identity with *Rickettsiella* strains described from *I. ricinus*. Analysis of *Rickettsiella* sequences from North American hard ticks showed that only a *Rickettsiella* spp. recorded in *I. angustus* was closely related to those of *I. ricinus* and clustered within Clade III. *Rickettsiella* sequences identified in *I. kingi*, *I. sculptus* and *I. woodi* gathered within a single clade that was distantly related to those observed in *I. ricinus* (Fig. 1).

This classification relied upon the genetic structure observed in parallel at nucleotide and amino acids (AA) levels. Overall, *Rickettsiella* populations within the same clade consistently experienced a low degree of variability since the uncorrected *p*-distance (*up*) averaged around 0.77% (min.-max. = 0–2.68; Table S1, Supporting Information). For instance, Clade I constituted the most conserved clusters, despite being composed of *Rickettsiella* from different geographic areas and harbouring sequences obtained from different arthropod species. Clade I-*gidA* sequences are closely related to *Candidatus* Rickettsiella isopodorum reported in terrestrial isopods species (JQ679309; JX406182) from Germany, sharing a high level of identity (> 95%). *Rickettsiella*-clades differentiation was sustained not only by the genetic distance, which showed mean values below 13.6% (Table S1, Supporting Information) among groups, but also by the AA composition they encoded. Using a 241 AA sequence of *Coxiella burnetii* as a reference, we could identify a total of 28 variable sites in the *Rickettsiella* AA sequences, which enabled us to determine the protein sequence motifs that characterized the single clades (Table S2, Supporting Information). The *gidA*-AA sequence profiling mirrored the genetic variability already observed within each *Rickettsiella*-clades. Clades I and II showed the highest degree of conservation since only an *Ala*–*Thr* substitution was detected, occurring at positions 140 and 52 of the reference sequence, respectively. By contrast, we detected a larger number of substitutions within Clades III and IV; in particular, sequences clustered into Clade III displayed *Ala/Thr*–*Pro/Ser*–*Lys/Asp/Glu*–*Tyr/His* substitutions at positions 13–80–124–148, while Clade IV exhibited *Ala/Ser*–*Glu/Lys*–*Lys/Arg* substitutions at positions 72–115–152.

**Fig. 2:**
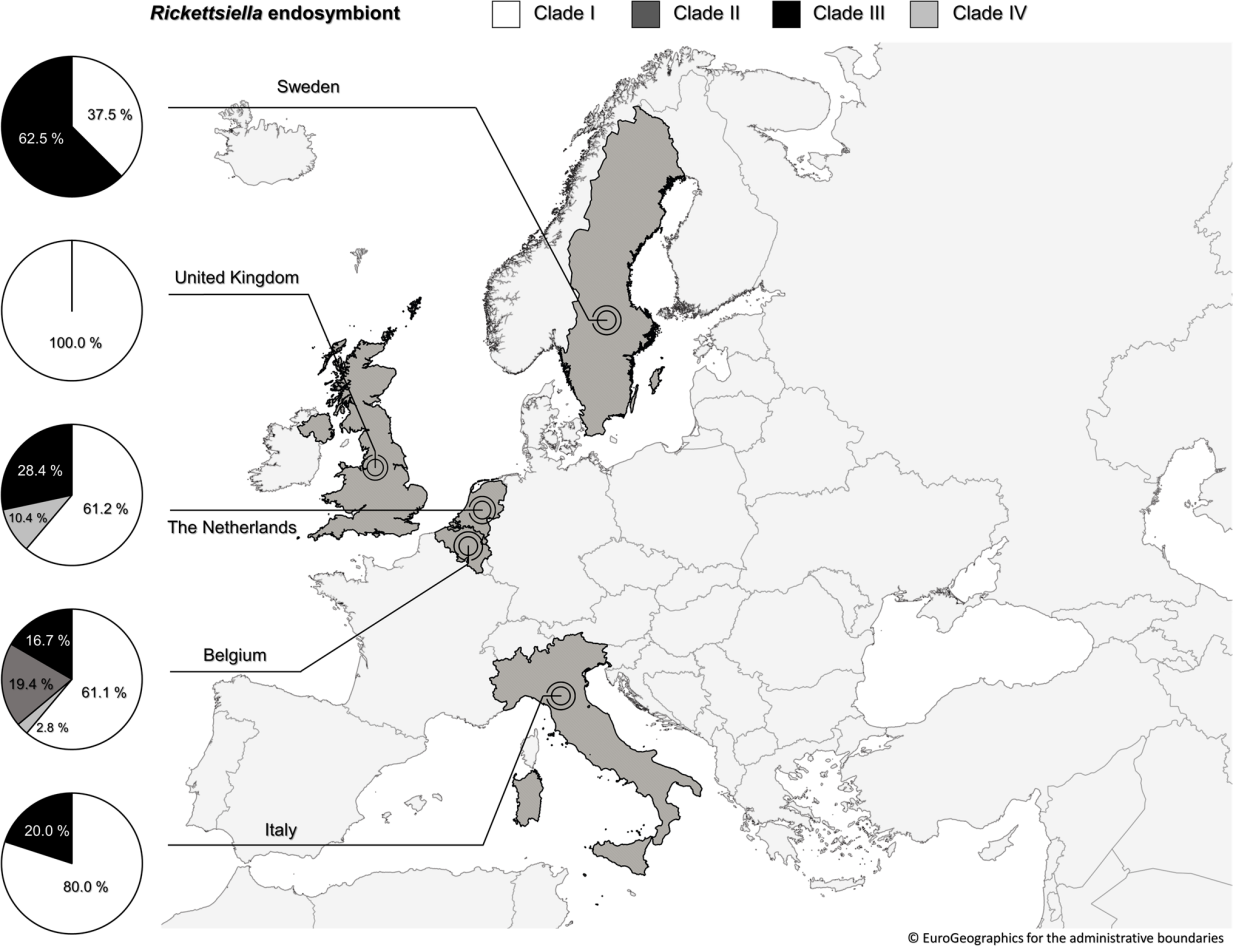
Geographical distribution and proportion of *Rickettsiella*-clades (I–IV) identified in *I. ricinus* populations from Belgium, Italy, the Netherlands, Sweden, and the UK

From a geographic point of view, different distribution patterns of each *Rickettsiella* clade were seen (Fig. 2). The most prevalent population, Clade I, was distributed among all *I. ricinus* investigated and was the only *Rickettsiella* clade detected in the British study sites. *Rickettsiella*-Clade III was detected in all study regions except for UK and prevailed in *I. ricinus* from Sweden. These two clades concurrently circulated with *Rickettsiella*-Clades II and IV in *I. ricinus* from closely related north-western regions. Clade II was only detected in ticks from Belgium study sites, whereas Clade IV was discovered in tick populations from the Netherlands and, to a lesser extent, Belgium. *Rickettsiella* spp. from Clade IV were closely related (95% identity) with *R. agriotidis* (JN565687), *R. costelytrae* (JN565689), and *R. pyronotae* (JN565691) that were recorded in different beetle species from Germany and New Zealand.

### Relationship of* Rickettsiella* spp. with Other Tick-Borne Microorganisms

Both investigated tick-borne pathogens, *B. burgdorferi* s.l. and *B. miyamotoi,* were present in all study areas. The prevalence of *B. burgdorferi* s.l. varied among the studied tick populations and geographic locations (Pearson’s chi-squared test, *p* < 0.001), with Sweden and Belgium showing the highest infection prevalence (Table 2). *Borrelia burgdorferi* s.l. infection prevalence was associated with the tick life stage. It was significantly higher in adult ticks compared to nymphs (*p* < 0.0001). The odds of *B. burgdorferi* s.l. infection for adults, in fact, doubled those for nymphs (OR = 2.1; 95% CI = 1.6–2.7). *Borrelia burgdorferi* s.l. prevalence in adult ticks in Belgian and Italian tick populations (29.8% and 38.1%, respectively) was also significantly higher than the prevalence in nymphs (16.8% and 14.5%, respectively; *p* < 0.001).

Neither geographic location nor tick life stage was associated with the occurrence of *B. miyamotoi* in ticks. The infection prevalence of *B. miyamotoi* was low in all studied areas (Table 2) and did not differ among regions (p = 0.07). The pathogen was observed in nymphs from all the investigated countries, but only in adults from Belgium (2.7%; 95% CI = 1.1–5.4) and Italy (1.7%; 95% CI = 0.06–12.6).

A generally high *M. mitochondrii* prevalence was detected (Table 2). It significantly differed among the regions (*p* < 0.001). Infection rates below 50% were recorded in Germany (36.4%; 95% CI = 24.9 – 49.1) and UK (48.7%; 95% CI = 42.3–55.3). *Midichloria* infection was not associated with the tick life stage (*p* = 0.38), although adults and nymphs showed different infection rates depending on the country where they were collected (Table 2). In regions for which information on the sex of adults was available (Belgium, the Netherlands, UK, and Italy), the odds of *M. mitochondrii* infection were significantly higher in female ticks compared to that in males (OR = 18.0; 95% CI = 7.8–48.4).

We detected the highest rates of bacterial coinfections in ticks from Belgium (around 75%), followed by the UK and Sweden (Table 4 and 5). While around 70–80% of coinfected ticks from Belgium and UK exclusively involved symbionts, over 75% of coinfections from the other countries involved at least one pathogen and one symbiont (Table 4). In Germany, only two out of 27 infected ticks were coinfected, with *B. burgdorferi* s.l. and *M. mitochondrii*.

**Table 4:** Overall coinfection rates in *I. ricinus* ticks according to the geographic areas of origin and to the tick-borne bacteria involved

Country	% Coinfection [95%CI]	Type of coinfection (%) [95%CI]					
		B – Bm	B – M	B – R	Bm – M	Bm – R	M—R
Belgium	75.6 [73.7–77.5]	0.7 [0.4–1.3]	20.7 [18.6–22.9]	22.1 [20.0–24.3]	1.9 [1.2–2.7]	1.9 [1.3–2.8]	94.1 [92.8–95.3]
UK	42.8 [35.3–50.5]	0 [0–1.0]	13.5 [6.7–23.5]	14.9 [7.7–25.0]	0 [0–1.0]	0 [0–1.0]	87.8 [78.2–94.3]
Germany	7.4 [0.9–24.3]	0 [0–1.0]	100 [15.8–100]	0 [0–1.0]	0 [0–1.0]	0 [0–1.0]	0 [0–1.0]
Italy	19.2 [16.7–21.7]	2.6 [0.9–6.0]	86.3 [80.6–90.9]	7.9 [4.5–12.7]	2.6 [0.9–6.0]	1.1 [0.1–3.8]	18.4 [13.2–24.7]
The Netherlands	15.3 [12.1–18.9]	2.8 [0.3–9.8]	15.5 [8.0–26.0]	59.2 [46.8–70.7]	4.2 [0.9–11.9]	11.3 [5.0–21.0]	31.0 [20.5–43.1]
Sweden	33.5 [26.6–41.0]	1.7 [0.04–9.1]	61.0 [47.4–73.5]	18.6 [9.7–30.9]	3.4 [0.4–11.7]	1.7 [0.04–9.1]	30.5 [19.2–43.9]

Note: B, *Borrelia burgdorferi* s.l.; Bm, *Borrelia miyamotoi*; M, *Midichloria mitochondrii*; R, *Rickettsiella* spp

We detected all possible combinations of coinfections. Coinfections with two bacteria were the most frequently observed in all regions. Belgium recorded the highest rates of *Midichloria*–*Rickettsiella* coinfections, affecting around 75% of the tick population. In countries where coinfections in ticks predominantly involved pathogens (*B. burgdorferi* s.l. and *B. miyamotoi*) in combination with symbionts (*M. mitochondrii* and *Rickettsiella* spp.), *B. burgdorferi* s.l. played the major role since it was far more prevalent than *B. miyamotoi*. Around 60–70% of the coinfections observed in Italy, Netherlands and Sweden were constituted by *B. burgdorferi* s.l. and at least one symbiont. As regards multiple coinfections, with more than two bacteria, the combination *B. burgdorferi* s.l.–*M. mitochondrii*–*Rickettsiella* spp. was the most prevalent. Coinfections with all four investigated bacteria were recorded in ticks from Belgium, Italy, the Netherlands, and Sweden.

The life stage of *I. ricinus* was not associated with the frequency of coinfections by two bacterial species, while multiple coinfections, with three bacteria or more, markedly prevailed in adult ticks compared to nymphs (*p* < 0.001).

Our results did not show significant associations between *Rickettsiella* spp. and *B. burgdorferi* s.l., *B. miyamotoi*, and *M. mitochondrii*. The highest *κ*-values obtained indicated a ‘very low agreement’ (*κ* = 0.0–0.20), and corresponded with the following positive associations: *M. mitochondrii* with *Rickettsiella* spp. in ticks from UK (*κ* = 0.15; 95% CI = 0.024–0.27), and *B. burgdorferi s.l.* with *Rickettsiella* spp. in Italy (*κ* = 0.11; 95% CI = 0.051–0.17).

## Discussion

This study contributes to the limited available knowledge about *Rickettsiella* symbionts in *Ixodes* ticks throughout Europe. Our results confirm the broad distribution of this heritable bacteria among different tick populations, encompassing a wide array of habitat types and climatic environments. In particular, we observed *Rickettsiella* spp. in geographically distant populations of *I. ricinus*, in which they displayed significantly different infection rates and highly diverse genetic features.

Intracellular bacteria of the genus *Rickettsiella* seem common in *Ixodes* ticks, though they have been also detected in metastriate tick species such as *Amblyomma* spp. and *Haemaphysalis* spp. [52], and soft ticks [32]. *Rickettsiella* spp. have been reported in *Ixodes* ticks from all over the world, including *I. kingi*, *I. sculptus*, *I. angustus, I. woodi*, and *I. scapularis* from North America [18, 21, 53]; *I. tasmani* from Australia [19]; and *I. uriae* from different locations of the northern and southern hemispheres [22]. In addition, *Rickettsiella* spp. have recently been detected in the tree-hole tick *I. arboricola* [54, 55]. To date, however, there are relatively few records of *Rickettsiella* infection in *I. ricinus* in Europe [20, 32–35].

The prevalence of *Rickettsiella* spp. in *I. ricinus* in our study varied across its geographic range. This finding is in line with previous large-scale studies concerning the polar seabird tick, *I. uriae*, in which *Rickettsiella* prevalence ranged from 3 to 24% [22], and the Canadian *I. sculptus,* where the infection rate averaged from 2 to 42%, according to the geographic location [21]. We recorded a higher prevalence in *I. ricinus* from western Europe, where *Rickettsiella* infection rate fluctuated between 48 and 89%. Lower prevalence rates were recorded for tick populations collected from the more northern and southern latitudes here investigated (16% and 3%, respectively). However, sampling differed in the studied countries, including different areas within the Netherlands, UK, and Italy versus unique sampling locations in Germany, Sweden and Belgium, and this could have influenced the detected prevalence.

Our results suggest that *Rickettsiella* spp. is a facultative symbiont of *I. ricinus*, and its infection rate varies depending on the environmental context. Also, the variable *Rickettsiella* infection rates in different tick species and populations might result from species-specific ecology and behaviour. For instance, *I. uriae* and *I. sculptus* generally live near their vertebrate hosts [36, 56], which might ensure the successful development and survival of ticks. In these circumstances, the cost of bearing a bacterial symbiont could outweigh the benefits potentially provided by the symbiont, leading to a low infection rate. Conversely, *I. ricinus* is exposed to a constantly changing environment [57]. The exophilic lifestyle hypothetically requires/facilitates relationships with facultative symbionts, which depending on environmental conditions, affect a variety of phenotypes of their hosts. Consequently, this could be one of the explanations for a significantly higher infection rate of *Rickettsiella* spp. observed in *I. ricinus* than in other tick species.

Moreover, environmental factors have been reported to affect the composition of microbial communities residing in *I. ricinus* [34, 35, 58]. Constant interactions between ticks and the immediate environment could lead to bacteria acquisition directly from the soil and vegetation [34, 58, 59], where they spend most of their life. Thus, differences in habitat characteristics might determine either the presence or absence of certain microbial taxa in different tick populations [20]. Accordingly, we may hypothesize that climate and habitat type could influence the spatial distribution of *Rickettsiella* spp., limiting its occurrence to local environments. For example, we observed remarkable differences in *Rickettsiella* distribution in mountain areas from the northwestern Alps: despite having similar habitat characteristics, we only detected the symbiont in one out of the two populations examined, in which 11.8% of the ticks were infected. Local microclimatic habitats might justify these differences. In fact, recent studies carried out in areas of the Swiss Alps reported a mean *Rickettsiella* prevalence of 63% in *I. ricinus*, uncovering a good correlation between tick abundance and precipitation with the occurrence of the bacterium [35]. However, we cannot rule out that the observed differences in *Rickettsiella* prevalence and diversity are stochastic events.

Essential biological processes of ticks, such as feeding and moulting, also affect the composition of tick microbiota [60] and may result in changes in microbial richness [58, 61]. In this study, we detected *Rickettsiella* spp. in *I. ricinus* larvae, nymphs, and adults, suggesting that the symbiont is maintained through successive life stages. Although no differences related to sex were observed, the prevalence was significantly higher in adults. As it has been indicated for other tick species [54], *Rickettsiella* sp. infection is possibly advantageous to ticks, increasing the survival success of infected individuals to the following life stage. Alternatively, the increased prevalence of *Rickettsiella* spp. in adults might result from an occasional horizontal transmission occurring, for example, via co-feeding of infected and uninfected ticks. Therefore, multiple feedings could increase the possibility of acquiring the symbiont. Nevertheless, in both scenarios, the subsequent transmission from adults to the offspring is hampered to some extent as we observe lower prevalence in larvae. To understand the transmission dynamics of *Rickettsiella* spp., future studies should investigate *Rickettsiella* sp. prevalence on a temporal scale and the role of vertebrates in propagating the symbiont in tick populations.

We detected an extensive genetic diversity of *Rickettsiella* spp. infecting *I. ricinus*. Four different clades composed the genetic structure of this symbiont, with some of them being associated with specific geographic areas. The diversity of *Rickettsiella* spp. was reported in other *Ixodes* species; for instance, Duron et al. [22] reported up to 12 genetically distinct strains in *I. uriae*, while three different 16S rRNA haplotypes were observed to coexist within *I. tasmani* populations [19]. On the other hand, only one *Rickettsiella* strain was reported for *I. angustus*, *I. sculptus*, and *I. kingi*, but in this study, the sample size was limited [21]. 

We acknowledge some limits with regard to *gidA* gene marker employed for this study. Relatively few *gidA*-reference sequences can be gathered from the GenBank database, thus preventing comparisons with *Rickettsiella* sequences already detected in other *I. ricinus* populations and in different arthropod species. Notwithstanding, *gidA* gene has been reported as a good marker to infer in the phylogeny of this genus, in particular at the infrageneric level [62]. *Rickettsiella* strains here identified, besides being genetically diverse, were also biologically different, as supported by the protein sequence motifs that defined each clade. These results may indicate that different *Rickettsiella* strains could have different roles in the life cycle of ticks. For example, it was suggested that *Rickettsiella* infection may affect tick reproduction in lab-reared colonies of *I. woodi* [18], while such effects were not related to the occurrence of *Rickettsiella* spp. in *I. arboricola* [55]. Moreover, some *Rickettsiella* strains have been historically considered entomopathogenic [23, 62], whereas other strains are facultative mutualist in their hosts [31, 63–65]. Whole-genome sequencing could possibly help in the identification and taxonomic classification of tick symbionts and in the evaluation of their biological relationship with ticks. For instance, this method could particularly help in clarifying the taxonomic attribution of *Diplorickettsia*, which was described as a new bacteria genus of *I. ricinus* [66], but is nested within the *Rickettsiella* genus [22, 67]. However, difficulties in isolation and cultivation still hamper the study of these microorganisms.

The close phylogenetic relations between *Rickettsiella* strains of *I. ricinus* and of other arthropods denotes the possibility of horizontal transmission among different arthropod species. Duron et al. [22] disclosed similar results in *Rickettsiella* strains identified in *I. uriae*, arguing that the symbiont might combine both vertical and horizontal transfers to ensure its maintenance and global distribution within arthropod communities. On the other hand, the specific natural environment, together with factors intrinsic to the tick hosts, might have significantly contributed to the evolution of *Rickettsiella* spp. In fact, isolation patterns were evident for some *Rickettsiella* populations: specific evolutionary forces experienced by *I. ricinus* and its symbionts may support the unexpected high variability observed in *Rickettsiella* populations from certain locations of central-western Europe. Consequently, further studies should investigate whether the genetic diversity of *Rickettsiella* spp. in *I. ricinus* accounts for adaptive selection in specific environments, while assessing potential co-phylogenetic associations between the symbiont and their tick hosts, as observed for other heritable endosymbionts [32, 68, 69].

Ticks are commonly co-infected with tick-borne pathogens, which have implications for public health [70]. The success of pathogen transmission, however, may be affected by their co-occurrence with symbiotic bacteria, leading to direct facilitation or competition effects among microbes [8]. Our results did not disclose any significant association between *Rickettsiella* symbionts and pathogens, which might entail that these symbionts do not interfere with the presence of *B. burgdorferi* s.l. and *B. miyamotoi*. These results are in line with previous studies in which a greater selection of tick-borne pathogens and symbionts was assessed [70]. By contrast, Aivelo et al. [35] reported strong associations between *Rickettsiella* spp. and different Lyme spirochetes. However, these positive correlations may be influenced by the greater proportion of adult ticks studied by Aivelo and colleagues, in which co-infections are more likely to occur compared to nymphs. Notwithstanding, our results do not exclude the possibility of associations between symbionts and pathogens. The heterogenicity of our results may not only be biased by differences in the epidemiological context, but also influenced by the diverse sample size tested per each geographic location.

## Conclusions

Our study demonstrates the widespread distribution of *Rickettsiella* symbionts among *I. ricinus* populations in Europe. Overall, *Rickettsiella* spp. displayed a high genetic variability according to the geographic locations, which highlights the importance of small-scale factors in shaping the distribution of this symbiotic bacterium. The in-depth evaluation of micro and macroclimatic variables affecting the occurrence of *Rickettsiella* spp. among different tick populations, as well as further phylogenetic studies, will hopefully elucidate co-evolution patterns between *Rickettsiella* spp. and ixodid ticks, and the specific geographic distribution of *Rickettsiella* strains. Moreover, our preliminary data suggest that *Rickettsiella* symbionts may also be present in other *Ixodes* species in Europe, but further research is needed to improve the knowledge about the symbiont’s ecology.

### Electronic supplementary material

Below is the link to the electronic supplementary material.

Supplementary file1 (PDF 231 kb)

## Data Availability

Representative sequence data are available on NCBI with Accession numbers: MW876489—MW876509.

## Appendix

**Table 5:** Origin and composition of the *Ixodes ricinus* populations investigated

Region	Location	Coordinates	Larvae tested	Nymphs tested	Adult tested	Males	Females
The Netherlands	Amsterdamse Waterleiding Duinen	52° 20′ N 4° 33′ E	244	103	1	0	1
	Bergherbos	51° 55′ N 6° 14″ E	-	51	5	2	3
	Buunderkamp	52° 00′ N 5° 44′ E	123	46	2	1	1
	Duin en Kruidberg	52° 26′ N 4° 36′ E	-	93	3	2	1
	Deelerwoud	52° 05′ N 5° 56′ E	-	56	8	3	5
	Herperduin	51° 45′ N 5° 36′ E	-	40	7	2	5
	Kremboong	52° 45′ N 6° 31′ E	-	76	12	10	2
	Stameren	52° 03′ N 5° 21′ E	-	45	3	1	2
	Zwanemeerbos	53° 00′ N 6° 45′ E	-	78	2	1	1
Italy	Alpi Cozie Natural Park	45° 03′ N 6° 54′ E	-	785	0	0	0
	Aosta Valley	45° 47′ N 7° 19′ E	-	257	39	22	17
	Tuscany	44° 12′ N 10° 22′ E	-	284	3	3	0
Germany	Güterfelde	52° 21′ N 13° 11′ E	-	66	0	0	0
UK	Salisbury	50° 59′ N 1° 45′ W	-	111	9	0	9
	Dartmoor	50° 34′ N 3° 55′ W	-	107	13	0	13
Sweden	Södermanlands	59° 00′ N 16° 52′ E	-	216	19	9	10
Belgium	Antwerp	51° 16′ N, 4° 23′ E	-	1771	262	136	126

Note: “-” indicates no specimens were tested for the corresponding tick life stage category
