# Supplementary material for: The Genetic Diversity of Rickettsiella Symbionts in Ixodes ricinus Throughout Europe
Source: Microb Ecol. 2021 Sep 28;84(2):613–26. doi: 10.1007/s00248-021-01869-7 (PMC9436858; doi:10.1007/s00248-021-01869-7)
Supplement: Supplementary file 1 — Supplementary file1 (PDF 231 kb) [file 248_2021_1869_MOESM1_ESM.pdf]

Table S1. Percentage of genetic variability (*up*) based on *gidA*–nucleotide and amino acids sequences within and among different *Rickettsiella* populations infecting *Ixodes ricinus*.

| | ***up* (%) [min. – max.]** | | | | | | | |
|---|---|---|---|---|---|---|---|---|
| | **Nucleotides** | | | | **Amino acids** | | | |
| **Clades** | **I** | **II** | **III** | **IV** | **I** | **II** | **III** | **IV** |
| **I** | 0.4 [0-1.7] | 13.2 [12.5–13.7] | 13.7 [11.3–14.8] | 14.3 [13.9–15.0] | 0.5 [0–2.8] | 7.4 [6.0–9.1] | 7.5[6.5–9.1] | 7.3 [6.6–9.1] |
| **II** | | 0.07 [0–0.3] | 11.7 [11.3–12.6] | 13.0 [12.2–13.5] | | 0.2 [0–0.8] | 7.5 [6.6–8.3] | 7.8 [7.1–8.7] |
| **III** | | | 0.58 [0–3.9] | 15.5 [14.5–16.0] | | | 0.5 [0–3.3] | 8.2 [6.8–9. 5] |
| **IV** | | | | 2.0 [0–4.8] | | | | 1.5 [0-3.3] |

Table S2. Protein sequence motifs of a fragment of *gidA* gene of *Rickettsiella* spp. obtained from questing *Ixodes ricinus* populations collected from different European regions.

| | 12 | 13 | 26 | 33 | 35 | 38 | 41 | 49 | 52 | 72 | 80 | 82 | 84 | 94 | 111 | 114 | 115 | 116 | 122 | 124 | 135 | 136 | 140 | 148 | 152 | 174 | 236 | 239 |
|---|---|---|---|---|---|---|---|---|---|---|---|---|---|---|---|---|---|---|---|---|---|---|---|---|---|---|---|---|
| **Clade I** | N | A | S | Q | N | V | M | Y | T | A | P | S | S | F | T | C | E | I | S | N | V | V | A | Y | K | V | A | T |
| | N | A | S | Q | N | V | M | Y | T | A | P | S | S | F | T | C | E | I | S | N | V | V | T | Y | K | V | A | T |
| **Clade II** | Y | A | S | Q | N | A | I | Y | A | A | P | A | S | L | T | Y | S | V | S | Y | R | D | A | Y | K | I | T | L |
| | Y | A | S | Q | N | A | I | Y | T | A | P | A | S | L | T | Y | S | V | S | Y | R | D | A | Y | K | I | T | L |
| **Clade III** | Y | A | S | E | D | V | V | C | T | A | P | A | S | L | T | Y | S | I | S | K | I | E | K | Y | K | V | A | I |
| | Y | T | S | E | D | V | V | C | T | A | S | A | S | L | T | Y | S | I | S | D | I | E | K | H | K | V | A | I |
| | Y | A | S | E | D | V | V | C | T | A | S | A | S | L | T | Y | S | I | S | E | I | E | K | H | K | V | A | I |
| **Clade IV** | K | T | G | Q | N | V | V | Y | A | A | P | A | F | L | S | F | E | V | S | N | I | E | T | Y | K | I | A | T |
| | K | T | G | Q | N | V | V | Y | A | A | P | A | F | L | S | F | K | V | S | N | I | E | T | Y | R | I | A | T |
| | K | T | G | Q | N | V | V | Y | A | S | P | A | F | L | S | F | K | V | S | N | I | E | T | Y | R | I | A | T |

**NOTE:**

A = Alanine
C = Cysteine
D = Aspartic acid
E = Glutamic acid
F = Phenylalanine
G = Glycine
H = Histidine

I = Isoleucine
K = Lysine
L = Leucine
M = Methionine
N = Asparagine
P = Proline
Q = Glutamine

R = Arginine
S = Serine
T = Threonine
V = Valine
Y = Tyrosine
